# “Moving on” through the locked ward system for women with intellectual disabilities

**DOI:** 10.1111/jar.12586

**Published:** 2019-04-05

**Authors:** Rebecca Fish, Hannah Morgan

**Affiliations:** ^1^ Centre for Disability Research Lancaster University Bailrigg UK

**Keywords:** forensic services, institutionalization, intellectual disabilities, recovery, secure settings

## Abstract

**Background:**

The move to community support for all people with intellectual disabilities is an aspiration with international significance. In this article, we draw on rich accounts from women with intellectual disabilities detained under the Mental Health Act (E&W) 1983 and staff at an National Health Service secure setting in England to explore how “moving on” is defined and perceived.

**Methods:**

The study reports on an ethnographic study using the field‐notes and the 26 semi‐structured interviews with detained women and staff on three wards.

**Results:**

We first explore staff conceptions of moving on, which include behavioural change and utilizing coping strategies. Then, we discuss the areas of analysis that women discussed: taking back responsibility, success in arranged relationships, acceptance of regime and resistance to progression.

**Conclusion:**

The concepts of moving on were not determined by the women but by the service. We recommend further research which explores women's own rehabilitation requirements.

## 1 INTRODUCTION

Despite (yet) another round of scandals concerning the treatment of people with intellectual disabilities in institutional settings in the UK leading to renewed policy commitments to the closure of such institutions (Department of Health, 2012; NHS England, 2015), a persistent number remains detained (Hatton, 2015,2016). The move towards individualized support is a global concern, with countries advancing at different rates (Beadle‐Brown, Mansell, & Kozma, 2007; Chapman, Carey, & Ben‐Moshe, 2014; Mansell & Beadle‐Brown, 2010). In terms of locked or secure inpatient units, people tend to have long lengths of stay (Alexander et al., 2015). This is because the process of relocation is lengthy and complex, involving multifaceted risk assessment and collaboration between services. The purpose of secure settings, also known as forensic inpatient services, is to provide assessment, treatment and care; with progression through services and rehabilitation viewed as key outcomes (RCP, 2013). Officially, people are placed within (UK) secure units if they are labelled as intellectually disabled and have committed an offence, or their behaviour is considered a risk to themselves or others (which may have led to a breakdown of a community placement). Most will be detained under the Mental Health Act (England and Wales) 1983, although some people may be detained using the Deprivation of Liberty Safeguarding process.

There is a small but growing body of research exploring outcomes from secure intellectual disability services (Alexander, Crouch, Halstead, & Piachaud, 2006; Alexander et al., 2011; Chester, Geach, & Morrissey, 2017; Morrissey et al., 2017), but qualitative reported outcomes mainly relate to male resident participants (the dominant group in secure services). This article presents findings from a study designed to explore the perspectives of staff and service users on three wards for women at a National Health Service (NHS) intellectual disability secure unit in 2012. Here, we explore the ways in which progression is conceptualized in services for people with intellectual disabilities and operationalized in professional practice on the unit. However, as we discuss later, the women themselves talked about “moving on” rather than “progressing” which tended to be used by staff and echoes the academic literature and policy and guidance documents.

### 1.1 Moving through services

Evidence of moving through, and from, institutional settings is important to both staff and service users (e.g., Long, Knight, Bradley, & Thomas, 2012). Existing literature about progression and rehabilitation generally focusses on particular empirical tools used to evaluate treatment programmes and analyse risk and recidivism rates in the longer term (Barron, Hassiotis, & Banes, 2004). There is very little research giving voice to users' views of potential outcomes from institutional provision (Chester et al., 2017). This is in sharp contrast to the literature around mental health rehabilitation, where the intention of services is to stimulate “recovery.”

Originating in the survivor movement, the concept of recovery has been adopted by activists as a way to describe the reclamation of a meaningful life (McWade, 2014). Theories of recovery in some therapeutic areas have moved away from the absence of mental illness symptoms to more subjective and holistic parameters (Schrank & Slade, 2007). Resnick, Fontana, Lehman, and Rosenheck (2005) used personal experiences in general psychiatric services in the United States to construct an empirical conceptualization of recovery and deduced four dimensions of recovery: the capacity to feel empowered in one's life; self‐perception and knowledge of one's condition; satisfaction with one's quality of life; and hope and optimism for the future. Mancini (2008) grouped together the themes conducive to recovery from a meta‐analysis of mental health literature and added the following to this list: autonomy and self‐agency, supportive relationships and enhanced role functioning. Mancini's work calls for a “self‐determination” model of recovery, which relies on three human needs (autonomy, competence and relatedness to others).

Although models of self‐determination are extremely relevant to the women in the present study, they are not easy to nurture in secure institutions, mainly due to the lack of real control and choice (Simpson & Penney, 2011; Turton et al., 2011). Compulsory care restricts liberty and autonomous decision‐making, particularly in settings for people with intellectual disabilities. People detained in secure settings are likely to have had traumatic past experiences, diminished community and family supports and therefore more complex service requirements (Simpson & Penney, 2011). Additionally, forms of peer support between service users are rarely encouraged (Clements, Clare, & Ezelle, 1995; Fish, 2015).

Recovery in secure services is more difficult to establish; however, Ward and Brown (2004) describe a model of recovery they refer to as the “Good Lives Model” of offender rehabilitation, replacing a focus on criminality and risk, with one which looks towards a future of well‐being and motivation. The person is included in discussions about their future, and the tasks they can fulfil to work towards recovery.

There has been comparatively little focus on this construct and its measurement within secure settings for people with intellectual disabilities. Morrissey et al. (2017) propose that in the context of intellectual disability forensic services, notions of recovery should incorporate the “connectedness; hope and optimism about the future; identity; meaning in life; and empowerment” (or CHIME) framework (Leamy, Bird, Boutillier, Williams, & Slade, 2011). Chester et al. (2017) researched patient defined outcomes in a forensic unit for people with intellectual disabilities and found that accessing therapies, experiencing good relationships with others, getting angry less often and learning skills for their move to the community were important to them. While these criteria are helpful for service providers, the authors' analysis does not show whether these are criteria defined by the service users or the service. The present article attempts to explore the origins of these notions and of the implications of the ways they are utilized on the wards for the women.

## 2 METHOD

This article has been drawn from an ethnographic research project with women with intellectual disabilities detained under the Mental Health Act (England and Wales) 1983 in locked wards (for a full description of methodology, see Fish, 2017b). 120 hr of observation took place on three wards for women in an NHS intellectual disability secure unit in England over a period of nine months. Women were a minority in the service, making up only 20% of service users. The service allocated people to a gender category, and all the participants were treated as women in the service. The ward managers designated which wards were to be observed. The wards were single sex and contained between two and eight women at any one time. Participant observation was used as a method to allow the researcher to find out what was important to women about their daily lives in order to develop an interview schedule. The themes which came out of the observation stage were as follows: relationships, power and control, “moving on” and future aspirations. The semi‐structured interviews included general questions about these themes and further prompts as necessary. The questions initiating discussion about this theme were as follows: What do you see for your future? How can you get to that point?

All participants in the observation phase of the research were invited to be interviewed, and although one member of staff and two residents declined, 10 staff and 16 residents agreed to be involved. All were white British and between the ages of 18 and 60. The staff interview participants were two male and eight female staff (seven qualified nursing staff, two unqualified support workers and one clinical psychologist). Each interview lasted between 8 min and 90 min, with some participants requesting follow‐up interviews.

### 2.1 Ethical matters

The research was given ethical approval from the National Health Service (NHS) Local Research Ethics Committee (Northwest Research Ethics Committee, code 10/H1016/138). All participants had the capacity to consent to participate in the research. Consent forms were used, with relevant checks for understanding in accordance with the Mental Capacity Act (England and Wales) 2005. All service users had information presented verbally in an accessible manner and were also given an information form to take away which was presented in pictorial and easier to read information to aid understanding.

### 2.2 Analysis

The interviews were transcribed, and at this point, they were anonymized, and participants were given pseudonyms. Together, the transcripts were analysed using NVIVO, which allowed the categorization of subthemes as well as facilitating comparison between groups for each theme. In this way, the analysis was inductive, arising from the data.

The results section below offers an analysis of the subthemes of the main theme “moving on,” namely: staff conceptions of progression, taking back responsibility, success in arranged relationships, acceptance of regime, and resistance.

### 2.3 Staff conceptions of progression

Progression held different meanings for staff and women on the unit, often depending on the reason for admission. However, staff and women acknowledged the necessity of progression for the women to be able to move on within and from secure settings. The overarching narrative was about enacting or producing some form of change in behaviour, typified by a staff member here:

Adele: It's not that you can change the person, you change the behaviour and the way they behave in a particular given situation, but first of all they have to recognise that that's how they behave in the given situation. And that's the hard bit, is getting people to actually say, 'Oh yeah, that's what I do and I don't want to do it any more and therefore I'll try and do this instead'. Once they've got to that point and they can really try, they may be able to then start learning better ways of coping so that they don't hurt themselves or others.

Although most of the staff described their perception of the women's negative past experiences as contributing to their current circumstances, this discourse left very little space to talk about positives in their lives, like good family and peer relationships, and skills and resources that women already had and could build on. There was evidence, however, that pasts were being used to contextualize behaviour and to work with the women to find out reasons, such as in staff member Stewart's account:

No matter what we do we're never going to cure people. The stuff that's–certainly in Joan's case–the stuff that's gone on, we're never going to get rid of and she'll never be okay with that, it'll always cause her problems. She's damaged now, unfortunately. As lovely as she is, her life is damaged by what's happened, and again, all we can do is make her feel safe, give her better coping strategies, but at times–those aren't going to work, so we've not to be too hard on ourselves when things go wrong. We'll go back to the drawing board, re‐design things again, get her involved, “Why did you hit that person?”

Stewart referred to evolving procedures, insofar as he was modifying his care in respect to Joan. He explained that the women's pasts were considered static and the women damaged as a result. Importantly however, Stewart demonstrated that keeping this knowledge in mind can sometimes be productive. Debate on this topic is divided: Adshead (2011) and Pollack (2007) consider looking to people's pasts to be problematic, because services' knowledge of past abuse adversely informs conceptions of risk. Conversely, other scholars advocate taking into account past trauma, but found that trauma is often not considered because services apply a medical model that merely deals with behavioural presentation (Brackenridge & Morrissey, 2010; Rossiter, 2012). A more holistic model would take people's pasts into account together with futures.

While staff views about progression focussed on behaviour, women's views were more diverse. When discussing their ideas about how they could “move on” in the service, three themes emerged: taking back responsibility, proving success in arranged relationships and acceptance of the regime on the unit. These were interrelated and complex, but all were models of progression which had been determined by the service and that the women engaged strategically with.

### 2.4 Taking back responsibility

Taking responsibility and gaining trust feature frequently in the literature about recovery in psychiatric services (Travers & Reeves, 2005; Turton et al., 2011), and these were also prominent in discussions about progression on the unit. For example, staff member Dawn talked about Jane who, after years of constant observation, was considered to be “progressing” extremely well and would soon be “moving on” into the community:

Dawn: We started off allowing her to go in the toilet on her own and we'd stand outside the door and just have voice contact with her. Very gradual, very slowly, and Jane can ask any time. She has two face‐to‐face contacts a day so she speaks to someone in the morning, and at night‐time about how she's feeling, whether she's settled, does she think she's well enough to be [unsupervised] or not?… So she's in control and it's really helped.

Rather than conceptualizing this as compelling women to obey the rules, Dawn described the process as one of handing over control. This process must have felt very risky at the time for staff. Jane herself spoke about this situation:

Jane: Well at first I got like five minutes in my room, then ten minutes, and in ward round I says “Can I have half an hour, just staff stay with me while I'm asleep.” I've gone off that, and then I've gone off [supervision] at daytime and I'm just off it now.

Although Jane stressed that she did not like being supervised constantly, most of the women desired the company of staff when they were not too busy; and they were reluctant to characterize this as supervision. Perhaps Jane's perceived dependence on the supervision was because she enjoyed the companionship, even though the accompanying surveillance felt punitive. Jane was slowly given more trust by staff and this worked because staff involvement was not significantly reduced at any point (as recommended by Turton et al., 2011). This is a good example of where treatment and security can co‐exist rather than being at odds with each other. Staff relinquished some of their control without negative consequences and Jane was seen to be self‐directing eventually, despite this being referred to as “taking control.”

### 2.5 Success in relationships

Elaine's perception of progression was being able to live successfully with another person. Elaine had lived by herself for a number of years. Elaine said that the reason she was in the unit was to “get better and move on”:

Researcher: How do you think you're moving on? What's helping?

Elaine: Well I am moving on now because I'm living with someone now, living with Teresa and I'm getting on alright with her all the time… [You have to just] prove that you can behave and prove that you can live with someone.

Elaine's perception of how she could move on was to “prove” that she had tolerance for living with another woman. She shared a ward with a woman who was described by staff as “resilient” and “laid back” in order to help Elaine learn to live with others. The progress that Elaine made was due to the sustained support from staff for both women throughout the process, and had been successful for a number of months, demonstrating that the stages of progression are individually planned.

Within this service, relationships between service users were not always encouraged when they became too close. According to Holland and Meddis (1993), this is because of the importance placed on staff/service‐user relationships. Indeed on this unit, positive staff/service‐user relationships were used as markers and facilitators of progress.

Some staff's concepts of progression tended to focus on absence of aggression and use of anger management techniques. However, they accepted that progression takes time. Staff member Monica, for example, focussed on relational factors as indicators of results, while noting that perhaps the reasons for lack of progression are more complex:

Women don't seem to move on very quickly. And I think with certain people you expect to see massive wins, and people get frustrated, 'What are we doing for her?' and I'll say, 'Well maybe she's not assaulted anyone for eight weeks, but you've not discharged her.’ Previously who was maybe assaulting someone every day. But they don't celebrate that.

Monica acknowledged that moving on is often a slow process, explaining how staff may feel when a woman is not seen to be progressing, but she also implicated the institution's organizational regime for this. Here, the omnipresent demand for progression on behalf of the service delivered power and control into the hands of staff. Monica construed progression in terms of service‐defined behavioural stability, a common interpretation in this unit. Although encouraging behaviour regulation in terms of aggression is likely to be beneficial, there is the danger of services promoting passivity if the context where the behaviour arises is not taken into account. Further, behavioural stability is a concept of progression which according to Alexander et al. (2011) is more often applied in women's services.

Describing positive relationships, staff member Monica used the word “trust”:

Monica: I think she managed to get quite a lot of good relationships on that ward for the first time ever, because that was around the time things started changing with the attitudes to self‐harm… We had a better approach and they began to trust us more, that we weren't going to lose our mind if they cut up [self‐harm] thinking we were going to get sacked. So things calmed a bit more, so I think the team on there were to thank for helping her to move on, no individual.

Here, Monica refers to a change in policy around self‐harm, which allowed acknowledgement of self‐harm as a coping strategy. She attributes the policy for the fostered trust and collaboration towards progression.

Support and reassurance offered by staff was considered by management to be thorough, yet this was not always talked about as a good thing. Some managerial staff considered the women to be too happy or feel too safe to want to move on and attributed any resistance to this, Karen for example acknowledged the importance of built relationships and women's reluctance to leave them behind:

[Women are not moving on] because of the relationships, and they feel safe. I mean would you want to go out there really? I mean you're here, you've probably made the first friends you've ever made, the staff are kind to you… And it's the trust and the relationships they make with the staff, they don't want to leave them.

This type of situation must constitute a dilemma for staff: good staff/service‐user relationships on one hand are discussed in positive terms, as keeping people safe, but on the other, as holding the women back from moving on. Karen's interpretation was that the institutional regime and the therapeutic relationship is too successful and can cause problems, yet did not acknowledge that the reason it is problematic is because support drops drastically in the less secure areas due to lower staffing levels. Her solution to this was to suggest making the regime *more *restrictive thereby emphasizing the independence women would experience after moving on.

### 2.6 Acceptance of regime

Another conception of progression involved notions of “acceptance” of staff decisions and the institutional regime. Staff member John spoke about Annie's recent progress where she had been more easy‐going when plans such as outings were cancelled or were changed. John was pleased about this, but like other staff, was concerned about the lack of ways to report positive behaviour due to the systems in place focussing on problematic ones:

I find that when they do the ward round reports they look at the trips out and the bad behaviours, because nobody has got the time to go through the daily notes. I personally think that when they've had an exceptional day it gets overlooked and maybe the people who do the notes who work it out could do a note or a flag up for it.

John felt there was an absence of time or opportunities to report and reflect on improvement, echoing Monica comments about the lack of “celebration” of achievements (see also Long et al., 2012) and reflecting the relentless focus on women's negative behaviours. There seems to be evidence of some sort of “double speak” here, where staff mention allowing women to “take control” yet “bad behaviours” are taken foremost as evidence that women should not have control. This echoes the carers’ opinions in Chester et al.'s (2017) study, that secure services place too much emphasis on “incidents.”

Most of the women talked about the future in terms of when they “get out” of the unit–Kate described her detailed plans for the future, and this led into a discussion about how she could “get out,” which again involved acceptance of the institutional routine:

Kate: We just have to do what the staff tell us to do because at the end of the day they've only got our best interests at heart. Do whatever they say, don't refuse to take our medications, do whatever we have to do, behave, don't go against our treatment and care plans by refusing medication or refusing to eat. Don't refuse work because that can delay you going even if you refuse your work that can delay you. Don't refuse work, go even if you don't like it until you can get it changed, stuff like that.

Researcher: So if you do all these things correctly, what's going to happen?

Kate: Well obviously we'll have to do all this for like three months. Non‐stop.

Researcher: And will they tell you that you're doing things good?

Kate: Yes they'll tell us that we're going in the right direction… And they will say to us every now and again the doctors, “You're doing well, carry on and you won't have long left.”

Kate's beliefs about progression included “doing everything right”: keeping to institutional rules such as complying with treatment, including medication, and making sure she goes to work even if she does not want to. The criteria she specified were reiterated by most of the women and do not contain any personal notions of progression, only enforced requirements. However, Kate went on to say that she felt that she was progressing herself, due to the therapy she had received helping her to deal with her past experiences of trauma:

Researcher: When you said you've got rid of all those bad things, how do you feel you've worked through them?

Kate: Yes, it took a hell of a long time but we have we've worked through it together… like it's in my mind now, I'm thinking about it now while I'm talking to you… No, it doesn't upset me any more, it doesn't upset me.

Most of the women did not have a clear idea of how their progress was measured and what they had to achieve in order to move on. Sometimes, it seemed that expectations were too high. In this revealing example, service‐user Tanya described how difficult it was to interpret:

Researcher: What's counted as a good day?

Tanya: When you don't do anything wrong. You have to be happy.

Researcher: What's “wrong”? Not shouting?

Tanya: Not being quiet either.

Researcher: You're not allowed to be quiet?

Tanya: You're not allowed to be quiet because they'll think you're “on one” [sulking or brooding].

Researcher: So how can you convince them you are having a good day then?

Tanya: You have to be talkative and happy.

Researcher: Right, that's quite a hard [task]...

Tanya describes the almost impossible situation of trying to convince staff that she is having a “good day.” A woman is therefore judged as moving on only when her demeanour fits a very narrow ideal “talkative and happy” on a regular basis (see also Webb, 1999). This is similar to a claim made by a participant in Goodley's study that people with intellectual disabilities are expected by staff to act “more normal than normal people” (Levine, cited in Goodley, 2001:215). Indeed, Sarah's concept of moving on also involved unrealistic acceptance of the institutional regime. She also described the difficulties involved with expectations of progression:

Researcher: And is there anything that you feel that you have to do to move on?

Sarah: Don't have any incidents… Like I got hit last week off [names service‐user]. And I just sat there and let her do it, I didn't want to hit back and that's why the staff said, “That's good that you didn't hit back.” So that's why they're going to move me on.

Sarah's idea of moving on was the absence of incidents, which aligns with model adopted by the majority of participants. However, Sarah points out that she was expected to accept an assault and not retaliate, and this was a sign to the staff that she was managing her anger. This is problematic when considering a future move to the community where safeguarding could be an issue if strategies of self‐defence have been eroded.

Staff member Wendy, however, did not see incidents as setbacks and acknowledged the role of staff in their onset:

Wendy: If you don't give them small goals then they're never going to go forward and they're never going to achieve what they want to do but [name], she achieved a hell of a lot in her time down there, she did really well. She had a few incidents like, but that's what, I think you should set the goals, and you might have an incident but you've got to learn from that incident and think you might have to do it differently the next time and do it another way round. That's what they did and she achieved a lot really.

Wendy advocated the use of “small goals” to encourage progress, based on individual needs. She recognized that incidents may happen but that they can be used as a learning opportunity–and that staff can avoid such incidents by becoming more focussed on the reasons why they happen and making adjustments. This contradicts the dominant idea that service users should learn to accept any sort of behaviour without retaliation. Wendy's articulation is of a way forward which is gradual and flexible, but which involves adequate staffing and input, an approach recommended by service users in the mental health literature (Turton et al., 2011).

Despite Wendy's comments, many service users described negotiating a system which encourages “playing the game” by signifying that the behaviour arises from personal will. This seemed to inhibit any personal notions of progression beyond that necessary to enable them to move on within the service.

### 2.7 Resistance to progression

Some of the staff talked about women sabotaging their progress, and thereby the opportunity to move on, by causing an incident just before they were due to move on:

Wendy: Well they don't like change do they? I've noticed that when they do come to move onto the next stage, that they will do something to destroy it because that's about their self‐worth… They're frightened, and probably [have] a lot of lack of confidence about moving on. (Interview, staff, unqualified).

Aitken and Noble describe these acts of sabotage as women being accustomed to feeling hated; therefore, negative power is the only power they are used to possessing (Aitken & Noble, 2001). This type of model implies that a way out of this cycle would be positive risk taking and sharing of power in positive ways. However, this needs to be carefully planned. Surrendering power too abruptly can cause people to lose confidence, as staff member Helen pointed out:

If we talk about moving people on and perhaps doing something to take a step back in the system, what used to be know locally as 'gate fever'. If you get anywhere near the gate you're feeling like anxious and they don't want to care for you any more and you've got to go out into the big wide world again and actually that's not a good place because horrible things have happened out there.

Any mention of sabotaging by the women, however, was discussed in purely strategic terms; for example, Kate talked about “playing up” so that she could move away from an unpleasant living situation after being moved to the step‐down service.

Kate: [In the step‐down service] There was a client there, it's not her fault it really isn't because she doesn't have control over her bowels or her bladder. But it always stunk of piss and shit, all over the place, all the time, and we used to say to the staff “Look, you need to do something with her because the thing is it's knocking us sick.” And they used to say, “It doesn't concern you.” And I used to say “Well actually it does because we're living like this.”

Researcher: Yes.

Kate: So I played up on purpose just to come back here.

Kate had made a decision to move back to the unit and managed to bring this about, which would no doubt have implications on the length of her overall stay in the service. Another woman mentioned that she had moved back due to an incident of aggression that happened over Christmas, which is a distressing time of the year for her:

Katrina: [I smashed my TV because] I was living in [step‐down service] and I had a bad time over Christmas so I had to come back. I wasn't well over Christmas. There were things going on with my family and they came to visit, and then there were problems with staff‐and I'm very sorry for what I did.

Researcher: I understand.

Katrina: I'll be going back to the houses [step‐down service] soon.

Staff: Yes well we're not sure about that at the moment. (Field‐notes).

Katrina knew she had disrupted her progress, and she was disappointed that she had been moved back to the unit. Women were moved backwards to a higher level of security for incidents such as this, and some women construed this as failure. This suggests that although staff may consider women to resist moving on because they are happy where they are, the women described that moving backwards was a negative result.

## 3 DISCUSSION

The results of this study suggest that institutional objectives can overshadow women's personal goals in terms of everyday life. Nevertheless, within the women's, and to a lesser extent, staff accounts we find subtle strategies of compliance and resistance that challenge the expectations and presumptions inherent in the structure and operations of the unit. In light of the Transforming Care agenda, where services are compelled to move people out of inpatient placements, this study has provided some helpful examples of how women can be supported to move on.

For secure services to be seen as therapeutic establishments, examples of women progressing through, and ultimately out of them, are essential. Progression and moving on was important to the women and was seen positively by the service, despite some staff accounts being at odds with this. Although it is reported in the literature that some service users fear moving on due to the loss of safety and security of the locked ward (Parry‐Crooke, Oliver, & Newton, 2000; Turton et al., 2011), these sentiments were not expressed by the women in the study and backwards moves were generally met with feelings of failure.

The criteria for moving on here, although planned using information about the individual, focussed on reduction of institutionally defined problematic behaviour. Travers observed a similar phenomenon in psychiatric services and claimed that this is due to women's admission to secure services as “determined by enduring behavioural disturbances in other residential environments” (2013:69). These behavioural disturbances are seen to require an environment with greater restrictions in order to modify them; therefore, behavioural stability determines progression (see also Aiyegbusi, 2002, Alexander et al., 2011). This may be to the cost of women's emotional well‐being, perhaps leading to strategies of self‐harm (Duperouzel & Fish, 2010).

This research suggests that personal notions of moving on were missing in favour of “proving” change to staff in ways some of the women found difficult to demonstrate. Tania clearly showed that to be considered “happy” she had to act within very strict parameters, and Sarah had to tolerate physical assault without retaliation in order to show she was eligible to move on. Hannah‐Moffat refers to the “neo‐liberal strategies of responsibilizing” (2000:528), where women are “empowered” to take responsibility for their actions, yet any failure to self‐govern results in more punitive supervision, thereby “*re‐enforcing”* existing relations of power (2000:529, emphasis added). This is the case here, the institutional regime negates any potential for empowerment and self‐direction.

A fundamental (service defined) concept of moving on discussed by participants was “acceptance”—of other people's behaviour, and of the institutional regulations and routine, including last minute changes to this regime (see Milton, Mills, & Jones, 2016 for an excellent critique of this expectation). Passively accepting aggression from other people and demonstrating it was possible to be able to live together with other service users was also key. This could encourage submissiveness and undermine the women's resilience when they leave the institution. By requiring service users to follow arbitrary rules, the system retains control while at the same time demanding evidence of responsibility being taken. Staff reflections imply that a way out of this cycle would be positive risk taking and sharing of power. However, this needs to be carefully planned as reducing support too abruptly can cause people to lose confidence.

The women had strong notions about their future including hopes for social integration, self‐sufficiency and (re)building family relationships (see Fish, 2017a), which challenge the institutional focus on their past experiences and current day‐to‐day conflicts. These themes overlap with the work of Chester et al. (2017), but were articulated in the present study as *despite* their stay in the service, rather than as outcomes of their stay.

What is missing in these accounts is how women wanted the service to help them achieve these future goals. Such accounts are visible in the recent mental health literature about recovery, even among those detained in forensic psychiatric services (Green, Batson, & Gudjonsson, 2011; Mezey, Kavuma, Turton, Demetriou, & Wright, 2010; Simpson & Penney, 2011). In the secure services literature, people want: self‐sufficiency, empowerment, life skills support and informal support networks for the future (Chester et al., 2017; Parkes & Freshwater, 2012; Richie, 2001). Given broader policy commitments to person‐centred practice (Department of Health, 2012) and well‐being as defined by the person—why are these criteria not used in concepts of moving on here?

The moving on that did occur was attributed to staff and service users working together towards shared goals. Although staff mentioned that individual planning by the multidisciplinary team did include objectives for each service user, this information was sometimes lost in the minutiae of daily incidents and control of the ward milieu (McCorkell, 2011). Focusing on service‐users' progression involves communication between staff on the ground and those making the plans. It also involves time and sufficient staffing levels, and for the organization to recognize successful therapeutic relationships as important springboards. Perversely, these successful relationships were occasionally misconstrued by staff as preventing women from moving on because service users become reliant on them.

It can be argued that with adequate and sustained support, as mentioned by Wendy and Jane, it was possible for women to move on without substantial setbacks. Key to this, however, was a sense of trust within the framework of close therapeutic relationships. McCorkel's research found that treatment techniques try to steer women away from dependency and emotionality, due to the idea that women's relational styles are deficient and irrational (McCorkel, 2003). It is considered possible to treat women as emotional and connected while supporting them to rehabilitate (McKim, 2008), but this is made difficult if relationships are construed as inherently problematic for them.

There is much evidence of good practice in the participants' accounts. Kate talked about how therapy had helped her come to terms with her past, and Jane discussed how she had learned to reduce her self‐harm, these were two personal outcomes that service users were pleased about. Although the main themes described could be considered akin to Mancini's measures of recovery (autonomy and self‐agency, supportive relationships and enhanced role functioning), these had not been articulated by the women themselves; for them, progress was established and measured through the eyes of the service. These practices could be described as encouraging passive compliance. Staff discussion often focussed on the women's pasts rather than futures (Adshead, 2010) and negative behaviours and efforts to challenge rules were sometimes regarded as a holding people back rather than being looked at contextually—indeed sometimes taking precedence over positive developments.

Snyder and Mitchell (2010:148) summarize our argument when they describe how these coercive ideas of progression typify institutional life in intellectual disability institutions and do not prepare people for any other:

Preparation, in the regulated life of institutions, does not prepare one for successful navigation of the outside world, for the structured regimes of institutional organization infiltrates the fibers of one's being. Thus, subjects become increasingly adapted to living a life whose parameters are no larger than the institutional grounds.

Looking towards a future in the community was extremely important to the women in this study (also shown by Chester et al., 2017) as well as being a human right most recently articulated in Article 19 “Living independently and being included in the community” of the UN Convention on the Rights of Persons with Disabilities (UN General Assembly, 2006). However, moving out of secure units is only successful if women are well prepared, and if there are sufficient and appropriate services in the community to support women who have been living in forensic services. By diverting the high levels of funding used to keep people in institutions towards community services, and by listening to people's experiences and involving them in their own plans, both custodial and community services can help people work towards their envisaged futures.

This study has limitations to generalizability. The findings are drawn from a single research site: three wards within one forensic unit in England. Discussions about progression were not an explicit focus of the research; however, the data analysis identified contested notions of progression as a central theme for staff and women. We recommend further research which explores women's own indicators of rehabilitation and moving on. Future research could also include talking to women after they have moved on from secure settings to find out the extent to which they were able to achieve the outcomes they (and services) wanted. It would also be interesting to know how their views may change after a period of time away from the secure setting. It would also be interesting to find out whether community‐based practitioners consider that these criteria for progression appropriately prepare women to return to the community. Additionally, more progressive therapeutic approaches than moving through secure wards such as the use of proactive or preventative community services and/or community sentences for offenders were not discussed, and we recommend that these approaches should be explored for women in particular.
